# PDA in Prematurity: Rethinking a Decades-Old Debate in 2026

**DOI:** 10.3390/biomedicines14030576

**Published:** 2026-03-04

**Authors:** Phoenix Plessas-Azurduy, Anie Lapointe, Sarah Spénard, Wissam Shalish, Marc Beltempo, Guilherme Sant’Anna, Gabriel Altit

**Affiliations:** 1 Division of Clinical & Translational Research, Department of Medicine, McGill University, Montreal Children’s Hospital, Montréal, QC H4A 3H9, Canada; phoenix.plessas-azurduy@mail.mcgill.ca; 2 Division of Neonatology, Department of Pediatrics, CHU Sainte-Justine, Université de Montréal, Montréal, QC H3T 1C5, Canada; anie.lapointe@umontreal.ca; 3 Division of Neonatology, Department of Pediatrics, Montreal Children’s Hospital, Montréal, QC H4A 3H9, Canada; sarah.spenard.med@ssss.gouv.qc.ca (S.S.); wissam.shalish@mcgill.ca (W.S.); marc.beltempo@mcgill.ca (M.B.); guilherme.santanna@mcgill.ca (G.S.)

**Keywords:** patent ductus arteriosus, prematurity, expectant management, pharmacological closure, nonsteroidal anti-inflammatory drugs, acetaminophen, bronchopulmonary dysplasia, postnatal corticosteroids, neonatal outcomes

## Abstract

The management of patent ductus arteriosus (PDA) in premature infants remains a significant debate in neonatology. Interventions aimed at accelerating ductal closure, often using nonsteroidal anti-inflammatory drugs (NSAIDs) or acetaminophen, are common practice. However, recent evidence increasingly challenges this approach. Pharmacological agents for PDA closure demonstrate limited efficacy and carry significant risks of systemic toxicity, affecting renal, gastrointestinal, vascular, and pulmonary systems. Multiple recent randomized controlled trials (RCTs) and meta-analyses have largely failed to demonstrate that early active treatment improves crucial clinical outcomes such as mortality, bronchopulmonary dysplasia (BPD), intraventricular hemorrhage (IVH), or necrotizing enterocolitis (NEC). Some studies even suggest potential harm, particularly an increased risk of BPD and mortality in vulnerable extremely preterm infants. Procedural closure methods (surgical ligation, transcatheter techniques), while achieving anatomical closure, also pose significant risks and lack evidence of improved clinical outcomes. Given the high rates of spontaneous PDA closure, especially in extremely preterm infants, and the lack of proven benefit alongside potential harm from interventions, a paradigm shift towards expectant or conservative management is gaining support. This approach emphasizes supportive care, minimizing interventions, and may be complemented by the judicious use of postnatal corticosteroids in selected infants with significant lung disease, which might indirectly facilitate ductal closure by addressing underlying inflammation.

## 1. The Controversy Surrounding PDA Management in Premature Infants: Should We Accelerate Ductal Closure?

The management of patent ductus arteriosus (PDA) in preterm infants remains a highly debated topic in neonatology [1]. The terminology “attempt to accelerate ductal closure” rather than “treatment of the PDA” will be used in this review since it underscores the distinction between facilitating a physiological process versus attributing to the intervention the philosophical concept of managing a “pathological entity”. The term “treatment” carries implications of disease and therapeutic efficacy, yet the current evidence supporting PDA closure strategies is inconclusive and often conflicting. Recent literature reflects this ongoing controversy, with evolving studies frequently prompting the revision or abandonment of previously established protocols [1,2]. This review aims to challenge entrenched clinical beliefs and offers a perspective on where we currently are with PDA management in the context of prematurity in 2026. Specifically, our reassessment centers on the practice of early routine pharmacological closure, defined as systematic attempts to initiate ductal closure in the first days of life, as compared to the growing paradigm of individualized expectant or conservative management.

## 2. A Clinical Scenario and the Left-to-Right “Significant” PDA

Consider the case of a 24-week gestational age (GA) infant presenting at day 4 of life with escalating oxygen requirements, low diastolic blood pressure, and bounding peripheral pulses. These clinical signs prompt an echocardiographic evaluation to assess for patent ductus arteriosus. Concomitant chest radiography reveals a “white-out” pattern, likely reflecting a combination of alveolar collapse, inflammation, edema, or transudate accumulation. This radiographic presentation exemplifies the pulmonary fragility commonly seen in extreme prematurity. Prematurity is inherently associated with heightened systemic and pulmonary inflammation [3]. The exposure of an extremely immature infant to exogenous oxygen at a developmental stage not intended for extrauterine life introduces a substantial inflammatory insult via significative oxidative stress amplified by the presence of reactive oxygen species. Interventions such as mechanical ventilation further exacerbate this risk, inducing ventilator-induced lung injury through alveolar overdistension and atelectotrauma. Compounding this is the antenatal inflammatory milieu frequently encountered in preterm birth—conditions such as chorioamnionitis, preeclampsia, severe intrauterine growth restriction (IUGR), preterm labor, prolonged rupture of membranes, and bacterial colonization all contribute to a pre-existing inflammatory surge at birth. These cumulative insults place the preterm neonate in a state of heightened vulnerability.

**Figure 1:**
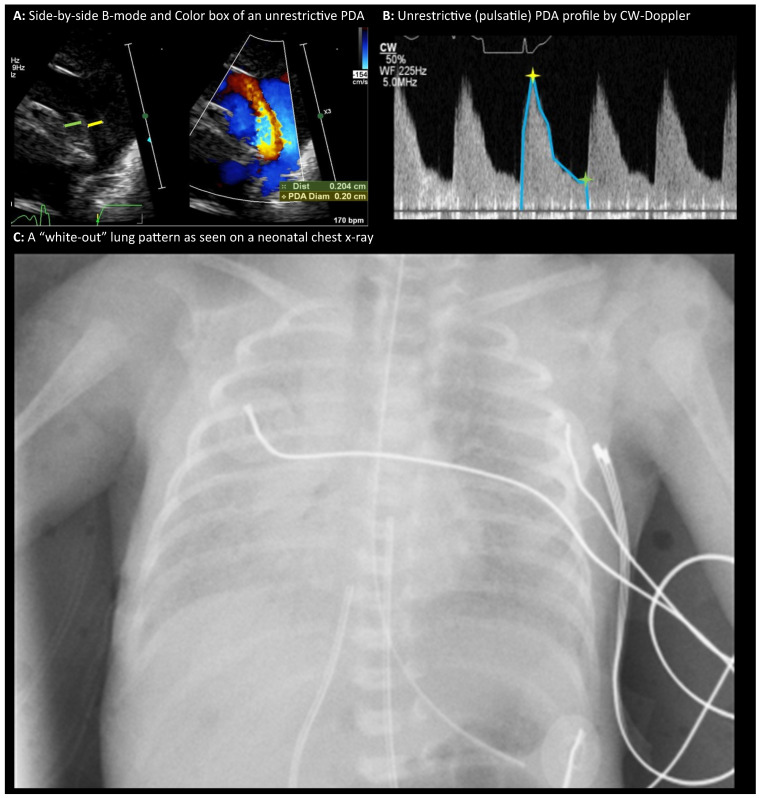
Diagnostic Imaging Profile of a Neonate With a 0.20 cm Unrestrictive PDA and “White-Out” Lung Pattern (**A**) Side-by-side B-mode and color box of an unrestrictive PDA with a 0.20 cm ductal diameter (in yellow) and left-to-right shunt (the left pulmonary artery is also measured alongside in green (0.204 cm) for comparison). (**B**) CW-Doppler profile of an unrestrictive PDA showing a pulsatile waveform with a sharp systolic peak (yellow star) and a diastolic decline (green star). (**C**) A “white-out” lung pattern as seen on a neonatal chest x-ray. Abbreviations: PDA (patent ductus arteriosus); CW (continuous wave); B-mode (brightness mode); cm (centimeters); bpm (beats per minute); MHz (megahertz); Hz (hertz).

Echocardiographic evaluation in our case may reveal left-to-right shunting from the aorta into the pulmonary artery via the ductus. Pulsed-wave Doppler interrogation of the ductus often demonstrates a pulsatile flow pattern in early life when the PDA is open and non-restrictive, characterized by a systolic peak followed by a diastolic decline. This waveform indicates the absence of a significant constriction along the ductal lumen (Figure 1). In cases of a non-restrictive ductus, low-velocity flow reflects minimal pressure gradient between the aorta and pulmonary artery, suggesting near-equal pressures and free transmission across the ductal segment.

This pressure equalization results in elevated pulmonary blood flow and pressure—physiologic conditions for which we are often worried that the immature pulmonary vasculature is ill-equipped. During fetal life, the large and patent ductus allows aortic and pulmonary arterial pressures to approximate, but pulmonary blood flow remains low due to the high pulmonary vascular resistance (PVR). After PVR drops in the post-natal setting, in a preterm infant with a persistently large PDA, the pulmonary vasculature prematurely experiences systemic pressures and high flow—conditions that deviate from the expected developmental trajectory. Excessive pulmonary blood flow through a PDA may lead to pulmonary congestion, especially in the context of a low-compliant left ventricle often seen in extremely preterm infant [4], increasing the risk of alveolar flooding and pulmonary edema. The resultant ventilation-perfusion (V/Q) mismatch is thought to compromise gas exchange, often prompting clinicians to escalate ventilatory support. However, increased mechanical ventilation can induce further lung injury and inflammation, setting up a cycle of worsening respiratory failure. Inflammation on its own triggers mechanisms that may maintain the PDA open and nonrestrictive, which is assumed by many to create a vicious cycle. This pathophysiologic sequence—initiated by ductal shunting and compounded by iatrogenic injury—has historically led to the belief that the PDA is a causative factor in the development of lung disease and complications such as pulmonary hemorrhage [5]. While the causality remains debated, the hemodynamic impact of a significant PDA in the fragile preterm lung warrants careful consideration.

### Arterial Steal and Retrograde Flow: The “Steal Effect”

As preterm infants progress through the first few days of life, PVR gradually declines [6]. In contrast, systemic vascular resistance (SVR) tends to remain relatively high. In the presence of a PDA, this imbalance facilitates left-to-right shunting, with augmented pulmonary blood flow occurring at the expense of systemic perfusion—a phenomenon often described as a “steal” effect.

**Figure 2:**
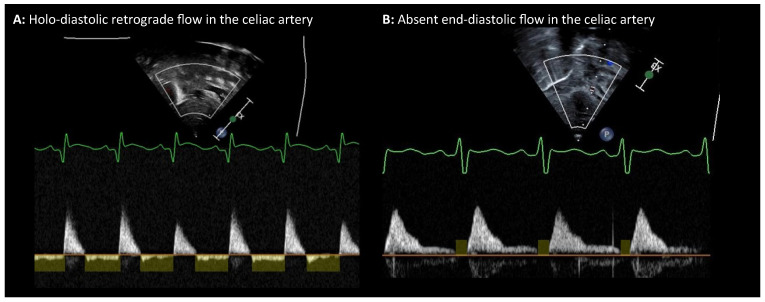

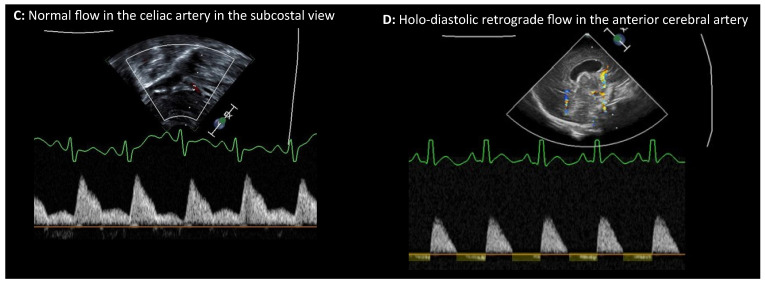
Doppler Flow Patterns in the Celiac and Cerebral Arteries (End-Organs) in the context of a “Diastolic Steal Effect” by the Left-to-Right PDA Shunt. (**A**) Holo-diastolic retrograde flow in the celiac artery obtained by PW Doppler in the subcostal view. (**B**) Absent end-diastolic flow in the celiac artery obtained by Doppler in the subcostal view. (**C**) Holo-diastolic retrograde flow in the anterior cerebral artery in the sagittal transfontanel views. (**D**) Normal antegrade diastolic flow in the celiac artery in the subcostal view serving as a reference pattern in a hemodynamically stable neonate.

Another key hemodynamic feature of an echocardiographically significant PDA is this so-called “steal.” In this setting, systemic blood flow is diverted during diastole from the systemic arterial tree—such as the descending aorta and splanchnic branches, as well as the cerebral arteries—back into the low-resistance pulmonary circulation via the PDA. This results in retrograde diastolic flow in the post-ductal/abdominal aorta, and, when profound, in some of the pre-ductal territories (middle and anterior cerebral arteries). It can be visualized using Doppler ultrasonography (Figure 2). The magnitude of this phenomenon is proportional to the ductal size and the systemic-to-pulmonary resistance gradient. Retrograde diastolic flow in cerebral arteries on cranial Doppler has been described as an alarming indicator of compromised cerebral perfusion (Figure 2).

**Figure 3:**
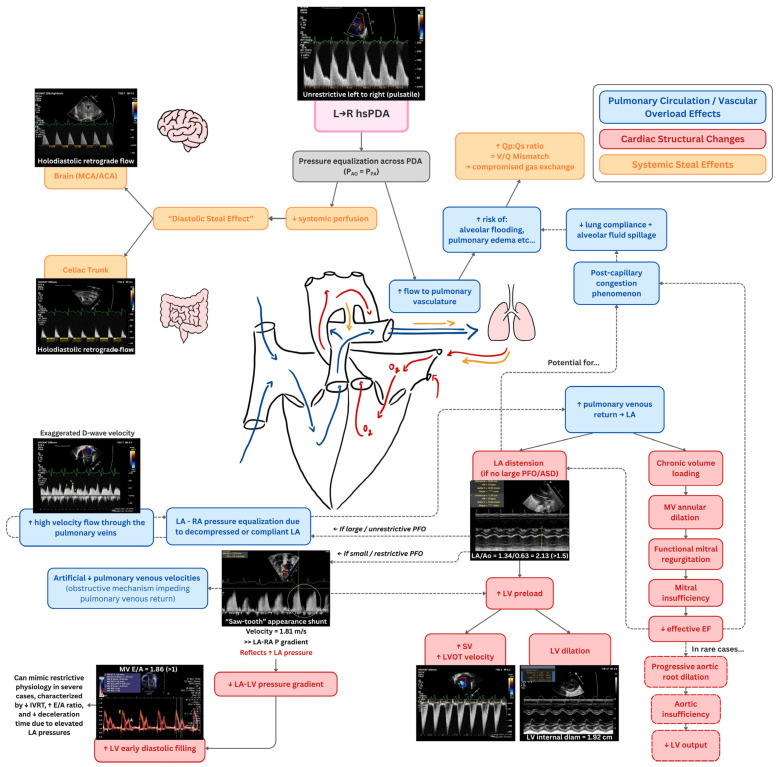
Conceptual framework illustrating the downstream cardiopulmonary consequences of a hemodynamically significant left-to-right PDA. Echocardiographic findings traditionally used to characterize hsPDA are presented here as manifestations of progressive pulmonary overcirculation, elevated left-sided filling pressures, and cardiac remodeling, rather than as diagnostic criteria themselves. Abbreviations: ACA (anterior cerebral artery); Ao (aorta); ASD (atrial septal defect); bpm (beats per minute); CW (continuous wave); E/A (early to late diastolic filling velocity ratio); ECG (electrocardiogram); EF (ejection fraction); Hz (hertz); LA (left atrium); LV (left ventricle); L→R (left to right); LVOT (left ventricular outflow tract); MCA (middle cerebral artery); mm (millimeter); MV (mitral valve); MHz (megahertz); PDA (patent ductus arteriosus); PFO (patent foramen ovale); PW (pulsed wave); RA (right atrium); SV (stroke volume); VTI (velocity time integral); Qp:Qs (pulmonary-to-systemic flow ratio); RV (right ventricle).

Additionally, in the case of a hemodynamically significant left-to-right PDA, excess pulmonary blood flow increases pulmonary venous return to the LA, leading to LA dilation when atrial decompression is limited by the absence of a large interatrial communication. In preterm infants with an immature, relatively noncompliant LV, progressive volume loading raises left-sided filling pressures and may contribute to post-capillary pulmonary congestion, impaired lung compliance, and alveolar fluid accumulation. LA distension, often reflected by an elevated LA/Ao ratio, typically evolves over the first days of life as pulmonary vascular resistance falls and pulmonary blood flow increases, with subsequent LV preload augmentation and chamber enlargement. Chronic volume overload may result in functional mitral regurgitation due to annular dilation, further elevating LA pressure and exacerbating post-capillary pulmonary hypertension; less commonly, aortic root dilation and aortic insufficiency may contribute to reduced effective forward flow and systemic steal. Doppler findings provide complementary physiologic insight, including restrictive left-to-right interatrial shunting, exaggerated pulmonary venous diastolic (D-wave) velocities reflecting increased venous return, and increased mitral E-wave velocities with shortened diastolic time intervals and inter-ventricular relaxation time in severe cases. These findings represent integrated manifestations of sustained pulmonary overcirculation, elevated left-sided filling pressures, and cardiac remodeling in hsPDA, rather than isolated diagnostic markers. Figure 3 depicts a conceptual framework of the downstream cardiopulmonary consequences of a hemodynamically significant left-to-right PDA.

## 3. Challenging Beliefs

In navigating PDA management, clinical decision-making should be informed by the best available evidence and an evolving understanding of ductal physiology. As knowledge advances, previously accepted practices may warrant reassessment, underscoring the importance of continually integrating emerging data into clinical care. In this context, some interventions aimed at ductal closure have shown limited benefit in certain populations and may carry potential risks, highlighting the need for thoughtful, individualized approaches grounded in current evidence.

Despite growing evidence questioning the efficacy of NSAIDs and acetaminophen, their use is still referred to as “treatment.” Yet these agents inconsistently achieve ductal closure and, more importantly, fail to improve meaningful clinical outcomes while exposing infants to potential toxicity. When closure does occur, it may simply reflect spontaneous resolution, a short-lived reduction in PDA caliber or treatment of lower-risk ducts rather than true therapeutic benefit. Persisting with ineffective or harmful intervention risks perpetuating an outdated paradigm. Progress will require moving beyond routine pharmacological closure toward more thoughtful strategies, including conservative or individualized approaches. Notably, the most immature infants—particularly those <26 weeks of gestation—often experience delayed but spontaneous ductal closure, in a median of 71 days [7], consistent with developmental reflecting their embryological programming to close the ductus only upon reaching a particular level of maturity that takes longer to achieve.

A fundamental and ongoing question in neonatology is not whether a hemodynamically significant left-to-right PDA produces measurable cardiovascular and pulmonary consequences, but rather the extent to which these consequences causally contribute to clinically meaningful adverse outcomes in premature infants. Specifically, which morbidities are directly mediated by ductal shunting and therefore potentially modifiable through targeted intervention, as opposed to reflecting the underlying vulnerability of extreme prematurity itself? Most PDA-directed therapies aim to close or significantly restrict ductal flow to reduce the hemodynamic burden imposed by the shunt; however, defining which outcomes are truly responsive to such interventions remains a critical challenge.

## 4. Physiologic Consequences and Disease Associations

In extremely preterm infants (e.g., 23–24 weeks of gestation), clinical interventions such as intubation and surfactant administration can precipitate a rapid drop in PVR, resulting in a surge of blood flow into the pulmonary circulation. Immature pulmonary capillaries, ill-equipped to accommodate such abrupt increases in flow, may rupture—culminating in pulmonary hemorrhage [8]. This may be particularly true if there is heterogenous vasodilation in the areas that the surfactant reached (ventilated), with some areas still vasoconstricted, limiting the redistribution of blood flow to areas with already lower vascular resistances. Additionally, hemodynamic transitions may destabilize perfusion across vulnerable vascular beds, including the splanchnic and cerebral circulations, particularly in the setting of impaired autoregulation. These are all speculative arguments brought forward for decades to justify the search of strategies to accelerate ductal closure. The cumulative impact of these hemodynamic perturbations—superimposed upon an already vulnerable developmental milieu—has been argued by many tenants of aggressive ductal closure strategies to contribute to the evolution of chronic pulmonary disease (bronchopulmonary dysplasia (BPD), retinopathy of prematurity (ROP), growth failure, pulmonary hypertension, and white matter injury) [9]. In the most severe cases, these complications can culminate in death. From a physiological standpoint, there is a compelling rationale that a persistently open duct may exacerbate or even precipitate these conditions.

### 4.1. Developmental Regulation of Ductal Patency and Closure

The high prevalence of PDA in extremely preterm infants is not coincidental; it reflects their developmental immaturity. In fetal life, ductal patency is maintained by a combination of elevated circulating prostaglandins—produced by both the placenta and the ductal endothelium—and relatively low oxygen tension [10]. As gestation progresses, the ductal endothelium becomes less responsive to prostaglandins, facilitating closure. Additionally, the anatomical substrate required for constriction—namely, a well-developed medial muscle layer—matures with advancing GA. At 23 weeks of gestation, this muscular component is often poorly developed, rendering the duct relatively unresponsive to stimuli for closure, even in the presence of elevated oxygen levels. The association between PDA and neonatal morbidities is further complicated by the multifactorial nature of many of these conditions.

NSAIDs or acetaminophen have been used for their property in modulating the prostaglandins expression or through the effect on peroxidase [11]. There may even be some speculation that these medications acutely increase PVR [12], as they are vasoconstrictors, which mitigate the trans-ductal shunt and promote closure. However, evaluating the impact of these interventions requires careful consideration of their systemic effects, the multifactorial nature of neonatal complications, and the confounding variables inherent to extreme prematurity. More importantly, their efficacy in both achieving closure in the desired patients and their capacity to mitigate the assumed complications of the duct are equally important.

### 4.2. PDA and Intraventricular Hemorrhage (IVH)

IVH in preterm infants arises from a convergence of antenatal and postnatal risk factors, including lack of antenatal corticosteroid exposure, outborn status, suboptimal resuscitation, absence of deferred cord clamping, and environmental instability at delivery [13]. Interestingly, prophylactic administration of indomethacin has been associated with a reduction in the incidence of severe IVH [14]. However, the benefit is unlikely to stem from ductal closure as its mechanisms of action include cyclooxygenase-mediated prostaglandin inhibition, reduced hyperemia from cerebrovascular hypoxia and hypercapnia, increased blood–brain barrier permeability, and protection against perfusion-related ischemia [14]. NSAIDs are potent systemic vasoconstrictors, exerting effects beyond the ductus arteriosus. Their influence extends to cerebral, renal, pulmonary, and mesenteric circulations. These drugs can significantly alter cerebral blood flow dynamics, potentially compromising perfusion to vulnerable brain regions [15,16]. This may occur for a prolonged period after exposure. Studies dating back to the 1990s demonstrated a sustained decrease in cerebral oxygenation following indomethacin administration, reflecting impaired cerebrovascular autoregulation and oxygen delivery [17]. Table 1 depicts a summary of the outcomes of intraventricular hemorrhage from key PDA trials.

### 4.3. PDA and Pulmonary Hemorrhage

While there is a concern that the left-to-right shunt from a PDA could overwhelm the fragile pulmonary vasculature and contribute to pulmonary hemorrhage, clinical trials have not shown that you can prevent pulmonary hemorrhage throughout hospitalization by aggressively attempting ductal closure. Rather, the hemorrhage is frequently the result of fragile pulmonary vasculature exposed to abrupt shifts in hemodynamic forces—particularly following interventions such as surfactant administration. Surfactant therapy, while essential for improving lung compliance and oxygenation, leads to a sudden drop in PVR. In the context of immature capillary networks, this rapid influx of blood can cause vascular rupture, even in infants without a PDA. Table 2 depicts a summary of the outcomes of pulmonary hemorrhage from key PDA trials.

An Australian randomized controlled trial (the DETECT trial) employed early echocardiographic screening and attempted to assess the benefit of early treatment [20]. Although it planned to enroll over 400 infants, the study was terminated early due to low recruitment and was underpowered for definitive outcome analysis. The trial recruited infants less than 29 weeks GA and did find that early indomethacin reduced pulmonary hemorrhage within the first 72 h (2% vs. 21%). Notably, the trial reported no significant reduction in pulmonary hemorrhage rates with early targeted (by echocardiography) treatment in the first 24 h of life (overall rate over the study period (9% vs. 23%, *p* = 0.07)) [20]. This observation suggests that the early reduction in pulmonary hemorrhage might be due to the direct pulmonary vasoconstrictive effect or anti-angiogenic properties of NSAIDs rather than ductal constriction itself. Although the difference in pulmonary hemorrhage rates between groups was not statistically significant, it is possible this was due to the trial’s small sample size since it was terminated early due to unavailability of indomethacin. The strikingly high pulmonary hemorrhage rate in the control group also raises the question of whether this finding was by chance. In comparison, in a cohort exposed to a strictly conservative management policy [25], pulmonary hemorrhage occurred in 21/280 infants (8%), an incidence below the reported one in the placebo group of the DETECT trial and similar to their indomethacin group.

The Trial of Indomethacin Prophylaxis in Preterms (TIPP) found no difference in pulmonary hemorrhage incidence between infants receiving prophylactic indomethacin and those receiving placebo (15% vs. 16%).

The TRIOCAPI trial reported that infants treated with ibuprofen had a lower incidence of severe pulmonary hemorrhage during the first 3 days compared to the placebo group (1.8% vs. 7.9%, *p* = 0.05) [21]. However, no significant difference between treatment groups was observed for overall pulmonary hemorrhage rates. Mortality rates were similar (mortality 20% (ibuprofen) vs. 14% (placebo), with a trend to be higher in the ibuprofen group).

Similarly, the BeNeDuctus trial reported a low pulmonary hemorrhage rate of 3% in the expectant management group versus 1% in the ibuprofen group, with no significant difference [22].

The NICHD-PDA Trial [26], a multi-center randomized clinical trial conducted across 33 centers in the United States randomized 481 infants to expectant versus active PDA treatment and reported death or BPD at 36 weeks PMA. They reported only 1 adverse event of neonatal pulmonary hemorrhage in the active treatment group (0 in the expectant management group).

Taken together, these findings challenge the hypothesis that the PDA is a primary driver of pulmonary hemorrhage. Instead, pulmonary hemorrhage appears to be influenced by a multitude of other factors, reflecting the complex immaturity and vulnerability of the preterm infant.

### 4.4. PDA and Necrotizing Enterocolitis (NEC)

NEC is a multifactorial disease, and while systemic diastolic steal from a PDA has been hypothesized to reduce mesenteric perfusion [27], autoregulatory mechanisms in some preterm infants may mitigate this risk by augmenting systolic ejection and left ventricular output. Nonetheless, pharmacologic agents themselves may contribute to intestinal injury. NSAIDs and acetaminophen (paracetamol) may exert direct gastrointestinal toxicity, particularly via prostaglandin inhibition and vascular constriction during exposure to treatment. Prostaglandins play a protective role in maintaining mucosal integrity, and their suppression may predispose to ischemic or inflammatory injury [28]. Additionally, the high osmolarity of oral formulations—especially acetaminophen syrup—may compromise the fragile intestinal mucosa in very low birth weight infants [29]. Table 3 depicts a summary of the outcomes of necrotizing enterocolitis from key PDA trials.

### 4.5. PDA and Bronchopulmonary Dysplasia (BPD)

BPD represents a chronic, multifaceted lung disease shaped by prenatal factors (e.g., chorioamnionitis, IUGR, placental dysfunction) and postnatal insults (e.g., oxygen toxicity, mechanical ventilation, infections, NEC) [30]. While excessive pulmonary blood flow from a significant PDA may theoretically induce pulmonary edema and inflammation, the underlying lung immaturity, impaired alveolar development, and inflammatory milieu are likely primary drivers. In fact, systemic inflammation itself can perpetuate ductal patency by stimulating prostaglandin production and endothelial activation [31]. The impact of postnatal corticosteroids on BPD adds another dimension. Trials such as PREMILOC [32] demonstrated that systemic low-dose hydrocortisone reduces BPD incidence, likely through anti-inflammatory effects. Interestingly, these improvements were accompanied by a decreased need for PDA intervention, highlighting the potential indirect benefit of reducing lung disease severity rather than directly addressing the PDA.

In the TIPP trial, infants without a PDA who received prophylactic indomethacin had a higher rate of BPD than those given placebo (43% [170/391] vs. 30% [78/257]). Multivariable analyses indicated that increased early oxygen dependence and greater first-week weight loss in the indomethacin group independently contributed to this elevated risk [18].

The 2025 NICHD-PDA Trial [26] found no difference in incidence of death or BPD at 36 weeks PMA between groups with a lower incidence of death by 36 weeks PMA in the expectant treatment group (4.1% (expectant) vs. 9.6% (active) *p* = X). Table 4 depicts a summary of the outcomes of bronchopulmonary dysplasia from key PDA trials.

### 4.6. PDA and Kidney Injury

Kidney injury in preterm infants may be long-lasting and could partly explain the association between BPD and systemic hypertension. NSAIDs such as indomethacin and ibuprofen are linked to acute kidney injury, reduced urine output and increased creatinine [34] with ibuprofen impairing renal function for weeks in very preterm infants [35]. Through systemic vasoconstriction and microcirculatory impacts, these drugs may contribute to renal and vascular remodeling [36]. Although long term effects remains uncertain, repeated NSAID courses—often given to the most vulnerable infants with persistent PDA—may increase the risk of renal injury and subsequent hypertension [37]. Similar concerns apply to pulmonary vascular injury (pulmonary hypertension), where NSAID toxicity could promote vascular remodeling and pulmonary hypertension, potentially confounding the observed associations between PDA and later cardiorespiratory outcomes. Furthermore, a critical limitation in the current evidence base for PDA management is the absence of long-term follow-up data regarding renal health beyond the neonatal period. While trials such as Baby OSCAR and BeNeDuctus have provided essential 24-month neurodevelopmental follow-up, similar longitudinal assessments of renal function are significantly lacking. Given that NSAID exposure in extremely preterm infants is associated with acute kidney injury and potential renal and vascular remodeling, these agents may have clinical consequences that persist into childhood and beyond. The collection of long-term data is therefore essential to fully understand the life-course implications of pharmacological PDA closure strategies on renal and cardiovascular health. Table 5 depicts a summary of the outcomes of kidney injury from key PDA trials.

### 4.7. PDA and Pulmonary Hypertension

As said earlier, NSAIDs may affect pulmonary vascular development by causing vasoconstriction [38], impaired angiogenesis, and reducing vascular density. Animal studies confirmed these adverse structural changes, and cyclooxygenase inhibition can increase pulmonary vascular resistance and promote pulmonary hypertension—effects that may also facilitate ductal closure [39,40]. Observational links between PDA and pulmonary hypertension are therefore highly confounded: the most vulnerable infant are both likely to have PDA and to develop lung injury and pulmonary vascular disease [41,42]. Similarly, we could associate BPD or pulmonary hypertension with other interventions commonly administered to the most immature infants, such as total parenteral nutrition (TPN), peripherally inserted central catheters (PICC), or gavage feeding duration, as they reflect the degree of immaturity rather than causality leading to pulmonary hypertension. Many cohorts reporting association of PDA with pulmonary hypertension routinely used NSAIDs, raising the possibility that medication-related vascular toxicity, rather than the ductus itself, explains the findings. In settings without routine NSAID exposure, pulmonary hypertension rate is similar [43], and it frequently occurs in infants without significant PDA. Another study noted that, despite the known risk of pulmonary vascular remodeling after prolonged PDA exposure in term infants, the duration of PDA exposure in preterm infants with BPD showed no measurable association with late pulmonary vascular disease during the neonatal admission, based on echocardiographic assessment [44]. Additionally, the NICHD-PDA Trial [26] reported only 1 adverse event of neonatal pulmonary hypertension in the active treatment group (0 in the expectant management group). Table 6 depicts a summary of the outcomes of pulmonary hypertension from key PDA trials.

## 5. Reassessing Pharmacological Closure Strategies

Management strategies for the PDA are generally categorized by the timing and indication for intervention: prophylactic, early targeted, and conservative (expectant) management. Prophylactic treatment involves the administration of pharmacological agents (typically indomethacin) within the first hours of life, regardless of clinical symptoms or echocardiographic findings, to prevent IVH or facilitate early closure. Early targeted treatment relies on echocardiographic screening within the first 24 to 72 h of life to identify a ‘hemodynamically significant’ PDA in asymptomatic or minimally symptomatic infants, with the goal of preventing later complications. Finally, conservative or expectant management prioritizes supportive care and allows for the natural evolution of the ductus, reserving intervention only for infants who demonstrate persistent, severe respiratory failure or hemodynamic instability later in the clinical course. While early active strategies aim to mitigate the perceived risks of prolonged ductal exposure, recent high-quality evidence from trials such as BeNeDuctus and Baby OSCAR increasingly suggests that these approaches may not provide the intended clinical benefits compared to a more conservative paradigm.

Current pharmacologic approaches to PDA closure, while grounded in decades of practice, warrant critical re-evaluation. As described earlier, the non-specific effects of NSAIDs and acetaminophen raise important concerns regarding organ perfusion and developmental toxicity. Furthermore, pharmacological attempts at closure have largely failed to demonstrate a consistent reduction in the duration of mechanical ventilation or the total length of hospital stay across major randomized controlled trials [21,45]. Procedural interventions including surgical ligation and transcatheter closure, are employed in clinical practice. Yet, the overarching question remains: are these interventions justified solely based on achieving ductal closure, or should their primary goal be to improve meaningful neonatal outcomes? This distinction is critical. If the intervention’s sole benefit is anatomical closure of the duct, without demonstrable improvement in morbidity or mortality—and if closure is accompanied by non-negligible adverse effects—then the rationale for intervention becomes tenuous. Studies have not definitively established that the intervention leads to a reduction in complications co-existent with PDA, such as IVH, NEC, BPD, or pulmonary hypertension, and whether it does so without incurring significant harm.

A large systematic review of 68 randomized trials including over 4800 neonates reported a pharmacological PDA closure rate of about 67% with indomethacin, ibuprofen, or acetaminophen [46]. However, spontaneous closure is common—particularly at higher gestational age—making it unclear how many treated PDAs would have closed without intervention and raising concerns about overtreatment [47]. Importantly, although these agents reduce ductal patency, meta-analyses [48] show no meaningful improvement in major clinical outcomes, questioning the clinical benefit of routine pharmacologic closure.

## 6. RCT Results

**Figure 4:**
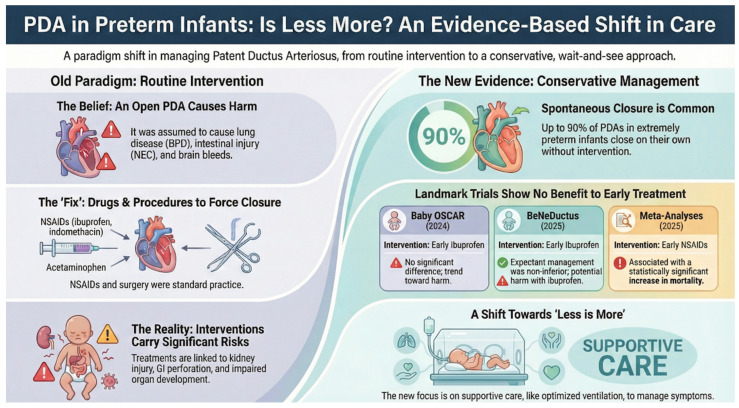
PDA in Preterm Infants: Is Less More? An Evidence-Based Shift in Care.

Multiple randomized controlled trials (RCTs), consolidated in Table 7, consistently demonstrate that early active PDA intervention does not significantly improve survival or reduce major morbidities compared to expectant management. Well-designed RCTs are structured to minimize biases and balance known and unknown confounders—factors that observational studies cannot fully control. As illustrated in Figure 4, the evidence from these major trials highlights a consistent lack of benefit for the composite outcomes of death or BPD, further supporting the shift away from routine early pharmacological treatment.

## 7. Reflecting on Real-World Practices and Entry Criteria

One criticism leveled against PDA trials, such as BeNeDuctus and Baby OSCAR, is their reliance on ductal diameter as a primary entry criterion. While diameter alone is acknowledged as an imperfect indicator of the full hemodynamic significance of a PDA (as outlined by the complexity of these evaluations in the sections above), there are several reasons why the approach used in these trials does reflect common clinical real-world practice for initiating treatment:

- Bedside echocardiography is routinely used to define the hemodynamic significance of PDA. A ductal diameter greater than 1.5–2.0 mm is commonly considered a significant PDA in most centers. Recent studies show that clinical practices in NICUs managing extremely preterm infants frequently involve treating a significant percentage of these babies (e.g., 54–73% of infants <27 or <32 weeks, 62–66% of those <25 or <26 weeks in the Canadian Neonatal Network) [50]. The criteria used in these trials yield similar rates of exposure than those described of current practices in most NICUs managing extremely preterm infants who use pharmacological interventions.
- Beyond Diameter Alone: Importantly, studies like Baby OSCAR did not rely solely on diameter. Baby OSCAR required a PDA diameter of at least 1.5 mm with unrestricted, pulsatile left-to-right shunting. This inclusion of flow pattern assessment makes the criteria more nuanced than just a simple diameter measurement.
- Magnitude of Shunt: The median PDA diameter in trials like BeNeDuctus was often well above the minimum 1.5 mm criterion, reported as a mean 2.1 mm. This indicates that the trials were enrolling infants with substantial shunts commonly targeted for treatment in clinical settings.
- BeNeDuctus: The trial reported a median PDA diameter of 2.1 mm at the time of the eligibility echocardiography.
- Baby OSCAR Trial: The entry criteria for the Baby OSCAR trial required a PDA diameter of at least 1.5 mm with unrestricted, pulsatile left-to-right shunting. For the babies included in the Baby OSCAR trial, it is notably reported that 75% of patients had a diameter of 2 mm or greater. The median diameter in this trial was 2.2 mm. Interestingly, 480 infants had a diameter above 2 mm vs. 166 infants with a diameter between 1.5 and 2 mm.

## 8. Representation of Extremely Immature Infants (<26 Weeks GA)

Another criticism is that PDA trials may not have included enough extremely immature infants, particularly those born before 26 weeks gestation. However, recent trials and subsequent analyses have made significant efforts to address this, a summary table can be found in Table 8):

- Recent trials, including Baby OSCAR and BeNeDuctus, enrolled a substantial number of extremely preterm infants. Baby OSCAR, for example, specifically reported outcomes in over 300 babies born below 26 weeks gestation (about half of their cohort were babies born at less than 26 weeks GA). Recent meta-analyses including trials conducted since 2010 found that these studies collectively included 707 infants born before 26 weeks gestation. According to the supplemental data from the BeNeDuctus trial, a total of 56 infants (<26 weeks GA) were in the Expectant Management group and 64 infants (<26 weeks GA) were in the Early Ibuprofen group at randomization, totaling 120 infants less than 26 completed weeks gestation. The subgroup analysis for infants born less than 26 weeks gestation at birth showed results for the primary outcome (composite of NEC, moderate to severe BPD, or death) that were consistent with the overall findings. The subgroup analysis for the primary outcome in infants born less than 26 weeks GA showed an absolute risk difference of −21.2 percentage points (95% CI −10.5). This indicates that in this subgroup, the primary outcome occurred 21.2 percentage points more frequently in the early ibuprofen group compared to the expectant management group, with the confidence interval strongly suggesting an increased risk (or harm) rather than benefit.
- Recent analyses pooling data from multiple trials that include substantial numbers of extremely preterm infants also failed to show a benefit and suggested harm from pharmacological PDA treatment in this population [1]. An analysis of seven randomized trials conducted since 2010 with low rates of open-label treatment, which collectively included 707 infants born before 26 weeks gestation, found that early treatment with NSAIDs to close persistent PDA increased mortality (pooled odds ratio 1.33, *p* = 0.05). This analysis also suggested increased risk for other morbidities like sepsis and PVL, and a trend towards increased BPD, pulmonary hemorrhage, and severe IVH [51].
- One review by Gupta S. et Donn SM [33]. described that data pooled from the El-Khuffash RCT, TRIOCAPI study, BeNeDuctus trial, and Baby OSCAR trial revealed a hazard ratio of 1.39 (95% CI: 1.05–1.78) for death by 36 weeks postmenstrual age in infants exposed to ibuprofen compared to those managed with placebo or expectant management.
- One meta-analysis from 2025 [2] included 10 randomized clinical trials involving 2035 preterm infants born before 33 weeks of gestation comparing active treatment (pharmacologic or surgical, though primarily pharmacologic within the first 2 weeks of life in the included trials) with expectant management for hemodynamically significant PDA. Active attempt to accelerate closure of a PDA during the first 2 weeks of life was associated with potential harm. Indeed, there was significantly higher rates of mortality (15.5% vs. 12.4%), with an odds ratio of 1.25 (95% CI, 1.01–1.56; *p* = 0.04). The meta-analysis also found a significantly worse composite outcome of death at 36 weeks postmenstrual age or at discharge or moderate to severe bronchopulmonary dysplasia (56.2% vs. 50.8%) (*p* = 0.009). The results were consistent in subgroup analysis for more immature infants born at less than 29 weeks gestation, where death was also significantly higher in the active treatment group (16.2% vs. 12.0%). The authors concluded that these findings suggest that an expectant management approach to PDA in preterm infants may be associated with a better morbidity and mortality profile.
- One Bayesian Meta-Analysis [52] included five RCTs. The study found strong evidence in favor of the harmful effect of medications regarding the outcome of BPD and for the composite outcome of BPD or death. When pooling the two largest trials (Baby-OSCAR and BeNeDuctus), the analysis showed moderate evidence in favor of higher mortality in the medication group. For the subgroup of infants ≤26 weeks GA, pooling data from Sung et al. and BeNeDuctus showed moderate evidence (Bayes factor +/− = 0.14) in favor of a higher rate of BPD or death in the treatment group. Including Baby-OSCAR for this subgroup also showed moderate evidence for a higher rate of BPD or death with medications exposure.
- Long-term follow-up data presented at scientific meetings for both the Baby OSCAR (PAS 2024 [45] and BeNeDuctus trials (ESPR) [53]) demonstrated no significant difference in survival without neurodevelopmental impairment (NDI) at 24 months of age. These findings are consistent with results from the TRIOCAPI trial and an observational study [54] comparing outcomes between infants managed under a strict conservative policy and those treated with ibuprofen to accelerate ductal closure.
- Of the 481 infants randomized in the NICHD-PDA Trial [26], 273 (56.8%) were born at 22–26 weeks gestational age. A prespecified subgroup analysis of these extremely preterm infants demonstrated no difference between expectant and active treatment strategies. Similarly, among infants with the largest PDAs (defined by the presence of at least one high-risk echocardiographic feature (ductal diameter >3 mm, ductal velocity <1.5 m/s, left atrium-to-aorta ratio >2:1, or diastolic flow reversal in the abdominal aorta)) there was no difference between treatment groups in the primary outcome of death or bronchopulmonary dysplasia at 36 weeks postmenstrual age.
- The TREOCAPA trial [55] included 803 infants born between 23 and 28 weeks of gestation, with specific dosing regimens tailored for the 23–26 week subgroup to ensure adequate assessment of efficacy and safety in the most immature patients. Despite this optimized pharmacological approach, the trial found that prophylactic acetaminophen did not increase survival without severe morbidity (defined as BPD grade 3, NEC stage II/III, IVH grade III–IV, or cystic leukomalacia) compared to placebo. Notably, the trial results suggest that even if early pharmacological intervention facilitates more frequent ductal closure, such anatomical success is clinically inconsequential as it fails to improve major neonatal outcomes or survival in this population.

## 9. Rethinking the Strategy for PDA in Prematurity

The accumulating evidence from recent RCTs highlights a paradigm shift in PDA management. Early pharmacologic closure—once considered a standard preventive strategy—is increasingly unsupported by data. These trials suggest that routine early treatment may not improve—and may in some cases worsen—key outcomes such as mortality (arguably the most critical outcome) and BPD. While BPD has its own limitations as an outcome measure and may not fully reflect what is most meaningful to families and patients in the long term [56], an increase in its incidence is nonetheless undesirable. Importantly, these interventions have not demonstrated any consistent neurodevelopmental benefit. Given the potential for harm and the lack of demonstrated efficacy, the default approach should no longer be automatic early closure. Expectant and conservative management may offer a safer alternative for many preterm infants, sparing them from unnecessary exposure to drugs with systemic toxicity and unclear long-term consequences.

## 10. Experience with Conservative Management: Observational Evidence and Evolving Practice

A retrospective review of local data from 2015 to 2019 provides interesting information regarding outcomes in a center with a conservative PDA management policy in extremely preterm infants [25]. This cohort, which was not exposed to NSAIDs or acetaminophen for PDA treatment, demonstrated high rates of spontaneous ductal closure. Specifically, approximately 90% of infants—regardless of whether they were below or above 26 weeks of gestation—achieved closure without pharmacologic or procedural intervention. These findings reinforce that even in the most immature infants, spontaneous closure is not only possible but common. Importantly, this conservative approach coincided with favorable clinical trends. To note, as part of routine respiratory management, the center made frequent use of postnatal corticosteroids, with many infants receiving dexamethasone courses. Reported cumulative doses fell within a low-to-moderate range overall, with median totals of 1.64 mg/kg (IQR 0.89–2.44) in the most immature infants and 0.89 mg/kg (IQR 0.89–1.20) in those born at later gestations (*p* = 0.01). Over the same period, a marked reduction in the composite outcome of death or BPD was observed. Mortality declined significantly, while BPD rates showed a downward trend—from 85% to 59% in infants under 26 weeks of gestation, and to 28% in those under 29 weeks. At 36 weeks corrected age, few infants remained ventilator dependent. The vast majority required only low-flow nasal cannula or CPAP, with invasive ventilation generally limited to those referred with primary airway disease. The unit ceased routine use of NSAIDs in 2013, citing concerns over toxicity and uncertain efficacy. In 2014, bubble CPAP became the primary modality for noninvasive respiratory support and the threshold for surfactant administration was set to 50% of FiO2 [57]. These practice changes correlated with a decrease in early intubation, BPD severity, and death from severe BPD. These positive and significant trends occurred alongside—and perhaps partly because of—the adoption of a strict conservative management policy of the PDA. Notably, they were observed despite a proportional increase in the number of infants born at 23–24 weeks of gestation and the inclusion of more fragile infants with birth weights near 500 g, who are typically at higher risk of inflating center-specific rates of adverse outcomes. While the data are observational and reflect multifactorial influences—such as improved nursing protocols, updated surfactant strategies, and transition to a new NICU facility—the key insight is that cessation of NSAIDs use did not result in increased rates of BPD or mortality. On the contrary, outcomes improved.

## 11. Acetaminophen (Paracetamol): A Limited and Possibly Harmful Alternative

Acetaminophen has emerged as alternative to NSAIDs for PDA closure, but its efficacy remains limited. In the PDA-TOLERATE trial [24], ductal closure occurred in only 27% of patients, with particularly poor results in the most immature. Safety concerns are also less well defined. While hepatic toxicity is recognized in adults, neonates exhibit high pulmonary cytochrome P450 activity, raising the possibility of localized pulmonary toxicity and oxidative stress [58,59,60,61]. Experimental and observational data suggest potential impairment lung development and BPD-like injury [60]. Additional concerns include the risk for cholestasis, and osmolarity of oral formulations. Which may contribute gastrointestinal injury, including spontaneous intestinal perforation—although this remains speculative and largely based on case reports and animal models [29]. The TREOCAPA trial [55], an international phase 2/3 trial involving 43 NICUs across 14 European countries, specifically addressed whether prophylactic acetaminophen increased survival without severe morbidity at 36 weeks postmenstrual age. The trial utilized a loading dose (20–25 mg/kg) followed by maintenance doses for 5 days, stratified by gestational age (23–26 weeks and 27–28 weeks). Ultimately, the results did not support the use of prophylactic acetaminophen for improving survival without severe neonatal morbidity, aligning with findings from NSAID-based trials that early active closure does not necessarily translate to improved clinical outcomes.

## 12. Procedural Closure: Surgical and Transcatheter Techniques

Procedural PDA closure including surgical ligation and transcatheter approaches provide definitive anatomical closure but carries substantial risks, and uncertain clinical benefit. Late intervention (e.g., at three weeks of life), is unlikely to influence early complications such as IVH or NEC while early procedures in extremely preterm infant (e.g., at two days of life in a 23-week infant) exposes them to significant harm, including post-ligation syndrome, cardiorespiratory instability, inflammatory responses [62], and long-term complications such as recurrent laryngeal nerve injury, diaphragmatic dysfunction, scoliosis, vocal cord paralysis, and (possibly) adverse neurodevelopmental outcomes [63] (although there might be a confounder by indication). Associations with BPD and retinopathy of prematurity (ROP) have also been reported. An RCT [64] of prophylactic ligation of the PDA in infants born at less than 1000 showed no improvement in survival with/without BPD or neurodevelopment, and later analyses suggested potential increases in respiratory and neurosensory morbidity. However, the study reported a significantly lower incidence of NEC in the ligation group (8%) compared to the control group (30%). The NEC (stage 2 or more) rate in the previously mentioned conservative cohort of extremely preterm patients <29 weeks was only 8% (*n* = 17/214), corresponding to the rate in the ligated group of the Cassady et al. trial [64]. In essence, the Cassady trial was one of the first rigorous attempts to evaluate a highly aggressive strategy (prophylactic ligation) for PDA in extremely preterm infants. While acknowledging the reduction in NEC, the trial highlighted the potential harms associated with early ligation regarding respiratory and long-term neurodevelopmental outcomes [65].

Transcatheter closure, though less invasive, still carries procedural risk—including vascular injury (e.g., limb ischemia, limb loss, hematoma, vessel rupture), atrial perforation, valvular trauma, device embolization, and mechanical obstruction of adjacent structures (aorta or pulmonary artery) [66]. Procedural anesthesia, bleeding, and the requirement for anticoagulation further increase risk. Critically, no study to date has demonstrated that transcatheter or surgical PDA closure improved clinically relevant outcomes such as survival, BPD, NEC, or neurodevelopment. Given these risks and the current absence of studies demonstrating benefit, the position articulated here is that procedural closure—particularly in fragile, extremely low birth weight infants—should only be undertaken within the context of a research protocol such as the PIVOTAL trial, (Percutaneous Intervention Versus Observational Trial of Arterial Ductus in Low Weight Infants—ClinicalTrials.gov ID: NCT05547165) following thorough parental counseling and informed consent. Offering invasive therapies without evidence of benefit and outside a trial framework is ethically questionable and potentially harmful.

## 13. Prioritizing Outcomes over Closure

Collectively, these data support a more conservative, physiology-driven approach to PDA management in preterm infants. Spontaneous closure remains the dominant trajectory for the majority of infants, even among those born at the limits of viability [7,25,67,68,69]. The rate of PDA closure following medical treatment in recent trials has been notably disappointing, achieving closure in only 50–70% of cases [70]. In comparison, spontaneous closure has been reported in 35–40% of extremely preterm infants [47]. Therefore, the actual difference in closure rates between treated and untreated groups is modest—only 18–35%, not 100% as is sometimes implied. Fundamentally, the physiology of ductal closure in preterm infants mirrors the developmental processes observed in utero. While more mature infants typically achieve closure earlier, even infants born at 23–24 weeks of gestation can close their PDA spontaneously over time. This delayed but natural closure trajectory has been documented across multiple cohorts [69].

## 14. Other Modalities: Postnatal Steroids

Supportive, non-interventional management remains a cornerstone of PDA care in many centers, particularly where the emphasis has shifted from routine closure to physiologically guided observation. One area of growing interest is the potential role of postnatal corticosteroids in promoting ductal constriction, either directly or indirectly through modulation of systemic inflammation. A study demonstrated that administration of dexamethasone around day 20 of life in preterm infants was associated with a reduction in ductal diameter, with some infants achieving complete closure [71]. Another study [72] found that postnatal betamethasone was associated with ductal constriction in extremely preterm infants and concluded that “In extremely preterm infants with a severe respiratory condition at 3 weeks of life, oral betamethasone treatment can help wean invasive ventilation and is associated with PDA closure. It could reduce the need for surgical or endovascular treatment that are associated with serious adverse effects.”

### 14.1. Animal Studies

Studies in fetal rats and lambs [73] have shown that steroid hormones, including dexamethasone, can cause ductal constriction in a dose-dependent manner. Potential mechanisms for how glucocorticoids like dexamethasone influence ductal closure include direct vasoconstriction, interference with prostaglandin (PG) synthesis (as PGE_2_ maintains patency), or a reduced sensitivity of the ductal muscle to the dilating effects of PGE_2_ [31]. Improvements in lung disease, increased diuresis, and improved oxygen tension may also indirectly facilitate ductal closure. Steroids can modulate inflammation, which may participate in keeping the duct patent.

### 14.2. Human Observational Data and Trials

Early dexamethasone therapy reportedly reduced the incidence of clinically detectable PDA in preterm infants weighing less than 1000 g at birth who had severe respiratory distress syndrome (RDS). One study reported an incidence of PDA of 23% in the dexamethasone group versus 59% in the placebo group (*p* = 0.05) in infants less than 1000 g [74]. This study also found no recurrence of ductal patency after indomethacin therapy in the dexamethasone group, compared to some recurrences in the placebo group.

Some older studies noted a temporal association between commencing dexamethasone therapy and PDA closure [75]. However, one older study on dexamethasone found no consistent closing effect on PDA in the four infants with PDA included in that particular analysis, noting that closure, when seen, often occurred at postnatal ages where spontaneous closure is common.

More recently, a prospective echocardiographic study evaluated low-dose dexamethasone (a 10-day tapering or ‘DART’ regimen with a cumulative dose of 0.89 mg/kg) to facilitate extubation or reduce respiratory support in infants with chronic lung disease (median age of 20 days to 30 extremely preterm) and found significant ductal constriction and closure in 35% of the cohort (7 out of 20 infants with PDA at baseline) [71]. The observed effect on PDA may be secondary to improvements in respiratory status or reductions in inflammation.

While NSAIDs and acetaminophen target ductal smooth muscle tone directly, corticosteroids may influence PDA physiology through systemic anti-inflammatory effects, potentially restoring the infant’s endogenous mechanisms for ductal closure [31]. In carefully selected cases—particularly those with evolving BPD, ventilator dependence, and inflammatory markers—postnatal dexamethasone may serve a therapeutic role beyond respiratory support, potentially aiding ductal closure and improving long-term outcomes. Nevertheless, spontaneous closure remains a reliable outcome in most infants when supported by evidence-based conservative care strategies.

### 14.3. Betamethasone (BTM)

Betamethasone has a vasoconstrictor effect on the PDA [76]. Studies in fetal rats showed increased constriction of the ductus arteriosus with combined administration of indomethacin and betamethasone [77]. Antenatal exposure to betamethasone has been associated with less frequent PDA in infants. The observed effectiveness of betamethasone could be related to its contractile effect on the ductus arteriosus. As mentioned above, a retrospective, single-center study of 51 extremely preterm infants [72] (median GA 25.7 weeks, median age at treatment 28 days) who received oral betamethasone to help wean from invasive ventilation found that BTM treatment was associated with PDA closure in almost all infants (98.0% had a closed or non-hemodynamically significant PDA after treatment). The percentage reduction in PDA diameter was significantly greater in the most immature infants (<26 weeks GA). The authors of this study suggest that this late oral BTM treatment could reduce the need for surgical or endovascular treatment, which are associated with serious adverse effects.

## 15. Systematic Reviews and Steroid Subtypes

A systematic review by Lex Doyle evaluated the effects of both dexamethasone and hydrocortisone on neonatal outcomes [78]. Hydrocortisone, though less effective in reaching pulmonary tissues, has value for cardiovascular support in the early postnatal period. Low-dose early hydrocortisone may confer some benefit in reducing BPD, although the confidence interval in pooled analyses crosses the line of no effect. In contrast, dexamethasone consistently showed a benefit in reducing BPD, particularly in trials where the baseline risk of BPD was high. This observation reinforced the notion that steroids are most beneficial in infants with severe inflammation—precisely the population at highest risk for adverse pulmonary and neurodevelopmental outcomes. Dexamethasone may therefore serve dual purposes: modulating lung and systemic inflammation and promoting ductal closure as part of a broader anti-inflammatory strategy. In their meta-regression analysis, “dexamethasone (compared with control) was associated with improved rates of survival free of cerebral palsy in infants at high risk of BPD but should be avoided in those at low risk”. This effect was not found in those exposed to hydrocortisone for pulmonary management [78].

Another study by Jensen et al. [79] described similar results in a multicenter cohort study of 482 matched pairs of infants born <27 weeks of gestation. They described the associated long-term outcomes of systemic corticosteroids (primarily dexamethasone) administered between days 8–42. Corticosteroid therapy was associated with a reduced risk of death or neurodevelopmental impairment at 2 years in infants with moderate to high pretreatment risk of death or severe BPD. However, in lower-risk infants, treatment was associated with potential harm, highlighting the need for individualized risk-based decision-making. Ultimately, while corticosteroids may indirectly facilitate ductal closure and provide significant respiratory benefits for infants at high risk for BPD, their potential for long-term neurodevelopmental impairment necessitates a cautious approach. They should be reserved for high-risk populations where the potential for reducing severe chronic lung disease clearly outweighs the risks associated with treatment, avoiding indiscriminate use in lower-risk infants where potential harm may exceed benefit.

## 16. Declining Treatment Rates and Evolving Conclusions

Over the past decade, PDA treatment rates—encompassing pharmacologic, surgical, and catheter-based approaches—have markedly declined across North America and the world [80]. This shift reflects a growing consensus that current interventions, despite extensive investigation, have failed to demonstrate consistent or meaningful improvements in clinical outcomes (and efficacy in achieving closure). At the same time, they are associated with a nontrivial risk of adverse effects. This evolving practice landscape prompts a fundamental ethical and clinical question: If an intervention for a physiological condition fails to improve outcomes and introduces harm, should it continue to be used? The weight of current evidence strongly suggests that pharmacologic strategies using NSAIDs, acetaminophen, or early procedural closure do not confer a survival or morbidity benefit in preterm infants. Numerous randomized controlled trials—despite involving thousands of patients and millions of dollars in research funding—have failed to show improvement in critical outcomes such as BPD, IVH, NEC, or neurodevelopmental impairment. On the contrary, these treatments carry significant risks, including renal dysfunction, gastrointestinal perforation, pulmonary toxicity, and impaired vascular development. Given this context, the routine administration of NSAIDs for the sole purpose of ductal closure—especially without explicit parental consent—raises serious ethical concerns. With overwhelming evidence pointing to a lack of benefit and the presence of harm, continuing to use these drugs indiscriminately is difficult to justify. Furthermore, additional randomized trials focused on these same interventions may also be ethically problematic unless they offer new hypotheses or alternative therapeutic frameworks.

## 17. A Shift Toward Conservative, Individualized Management

A large body of observational literature has consistently associated PDA with adverse outcomes in preterm infants; however, randomized trials to date have not demonstrated that strategies aimed at accelerated ductal closure reliably mitigate these risks. Future research should move beyond a binary open-versus-closed paradigm to evaluate novel, physiology-informed approaches—including modulation of ductal shunt magnitude and timing—while carefully balancing potential harms related to altered blood-flow redistribution, myocardial loading, and end-organ perfusion in the most vulnerable infants. Emerging data increasingly support a conservative, physiology-guided approach centered on supportive care and the avoidance of routine closure. Key elements of this strategy include:

- Non-invasive respiratory support, such as bubble CPAP, which reduces the need for intubation and mitigates lung injury and inflammation.
- Permissive hypercapnia to avoid inducing a drop in pulmonary vascular resistance.
- Avoidance of inhaled nitric oxide in the context of a left-to-right PDA.
- Vasopressors should not be administered solely to address isolated low blood pressure values—particularly when driven by low diastolic pressure—in the absence of clinical signs of organ hypoperfusion, such as rising lactate levels, decreased urine output, mottling, or prolonged capillary refill time.
- Standardized, high-quality neonatal care, including optimized nutrition, infection control, and family-centered support.
- Judicious use of postnatal corticosteroids, in infants with evolving lung disease and inflammatory markers, where inflammation may play a role in both BPD and PDA persistence.
- Fluid restriction, packed red blood cells or platelet transfusions, as well as induced diuresis are not recommended for the sole purpose of managing the ductus arteriosus.
- If a left-to-right PDA is still present at term-corrected age, its hemodynamic significance should be evaluated by echocardiography. The decision to then proceed with procedural closure or further follow-up must be done in conjunction with cardiology.

This approach is aligned with the foundational principle of neonatal care: less is often more. For these fragile and developmentally vulnerable infants, minimizing unnecessary interventions may result in better long-term outcomes. The burden of proof lies not anymore in demonstrating the presence of a PDA, but in showing that closing it improves outcomes without causing harm. The future of PDA management should prioritize long-term infant health over short-term anatomical correction, reserving intervention for cases with clear hemodynamic compromise or within well-designed clinical trials. At present, the most promising path forward appears to lie not in closing every duct, but in supporting every infant with a thoughtful, individualized approach combining advanced respiratory care and mitigation strategies to limit the shunt (such as mainly permissive hypercapnia which increased the PVR/SVR ratio and maintaining adequate non-invasive positive end expiratory pressure to allow lung growth and adequate functional residual capacity).

## Figures and Tables

**Table 1:** Intraventricular Hemorrhage Outcomes in Randomized Trials and Cohort Studies of PDA Management in Preterm Infants.

Trial	Design & Population	Intervention	IVH Findings	Notes/Interpretation
TIPP Trial [18,19]	Large multicenter RCT; 1202 ELBW infants (500–999 g)	Indomethacin prophylaxis vs. placebo	Severe IVH (Grade 3/4): 9% (Indomethacin) vs. 13% (Placebo), *p* = 0.02	Prophylaxis significantly reduced the incidence of severe IVH, though it did not improve long-term neurosensory survival.
DETECT Trial [20]	Early targeted RCT; <29 weeks GA; screened before 12 h	Indomethacin vs. placebo for large PDA	PIVH (Grade 2–4): 4.5% (Indomethacin) vs. 12.5% (Placebo), *p* = 0.21	Showed a trend toward reduction in brain lesions but was underpowered due to early termination.
TRIOCAPI Trial [21]	Double-blind RCT; <28 weeks GA; large PDA at 6–12 h	Early targeted ibuprofen vs. placebo	Grade 3/4 IVH: 15.8% (Ibuprofen) vs. 9.6% (Placebo), *p* = 0.20	Observed a non-significant trend toward increased high-grade IVH in the ibuprofen group.
BeNeDuctus Trial [22]	Noninferiority RCT; <28 weeks GA; PDA >1.5 mm	Early ibuprofen vs. expectant management	IVH Grade ≥ III: 6.6% (Ibuprofen) vs. 8.1% (Expectant)	Rates were similar between groups; pharmacological treatment did not provide protection against severe IVH.
Sung et al. Trial [23]	Single-center RCT; GA 23–30 weeks	Oral ibuprofen vs. nonintervention	IVH Grade ≥III: 3% (Ibuprofen) vs. 6% (Nonintervention)	No significant difference observed (adjusted OR 0.50, 95% CI 0.08–3.25).
PDA- TOLERATE Trial [24]	Exploratory RCT; <28 weeks GA; enrolled at 6–14 days	Early routine treatment (ERT) vs. conservative (CT)	Serious IVH (Grade 3/4): 18.3% (ERT) vs. 11.2% (CT)	Early treatment did not reduce severe IVH and trended higher in the treatment group.

Abbreviations: GA (gestational age); PDA (patent ductus arteriosus); RCT (randomized controlled trial); ELBW (extremely low birth weight); IVH (intraventricular hemorrhage); PIVH (papillary/intraventricular hemorrhage); TIPP (Trial of Indomethacin Prophylaxis in Preterms); TRIOCAPI (Trial on Ibuprofen in Closure of Arterial Patent Ductus); BeNeDuctus (Expectant Management or Early Ibuprofen for Patent Ductus Arteriosus Trial); ERT (early routine treatment); CT (conservative treatment); OR (odds ratio); CI (confidence interval).

**Table 2:** Pulmonary Hemorrhage Outcomes in Randomized Trials and Cohort Studies of PDA Management in Preterm Infants.

Trial	Design & Population	Intervention	Pulmonary Hemorrhage Findings	Notes/Interpretation
DETECT Trial [20]	<29 weeks GA; early ECHO screening; underpowered (N < 400)	Early indomethacin vs. placebo	- PH ↓ in first 72 h: 2% (indomethacin) vs. 21% (placebo) - No significant difference in PH over full study: 9% vs. 23%, *p* = 0.07	Suggests early PH reduction may relate to NSAID vasoconstriction or anti-angiogenic effect rather than PDA closure
Conservative Cohort [25]	Infants managed without PDA treatment (N = 280)	Expectant/ conservative management	- PH incidence: 8%	Lower than placebo arm in DETECT trial; similar to indomethacin group
TIPP Trial [18,19]	Large RCT; prophylactic indomethacin in preterms	Indomethacin prophylaxis vs. placebo	- PH incidence: 15% (indomethacin) vs. 16% (placebo)	No significant difference; argues against PDA closure as sole PH driver
TRIOCAPI Trial [21]	Ibuprofen treatment vs. placebo	Early ibuprofen	- Severe PH in first 3 days: 1.8% (ibuprofen) vs. 7.9% (placebo), *p* = 0.05 - No difference in overall PH rates	Trend toward early protection; overall rates similar; mortality higher in ibuprofen group (20% vs. 14%)
BeNeDuctus [22]	Expectant vs. ibuprofen	Expectant management vs. ibuprofen	- PH incidence: 3% (expectant) vs. 1% (ibuprofen)—not significant	Very low PH rates overall; difference not statistically significant
NICHD-PDA Trial [26]	Multicenter RCT (33 U.S. centers); 481 preterm infants randomized	Expectant management vs. active PDA treatment	- 1 case in active treatment group; 0 cases in expectant group	No difference in death or BPD at 36 weeks PMA; lower mortality with expectant management (4.1% vs. 9.6%), reinforcing lack of benefit and possible harm of routine active PDA treatment

Abbreviations: GA (gestational age); ECHO (echocardiography); PH (pulmonary hemorrhage); NSAID (non-steroidal anti-inflammatory drug); PDA (patent ductus arteriosus); RCT (randomized controlled trial); TIPP (Trial of Indomethacin Prophylaxis in Preterms); TRIOCAPI (Trial on Ibuprofen in Closure of Arterial Patent Ductus); BeNeDuctus (Expectant Management or Early Ibuprofen for Patent Ductus Arteriosus Trial); NICHD-PDA (National Institute of Child Health and Human Development Patent Ductus Arteriosus Trial); BPD (bronchopulmonary dysplasia); PMA (postmenstrual age).

**Table 3:** Necrotizing Enterocolitis Outcomes in Randomized Trials and Cohort Studies of PDA Management in Preterm Infants.

Trial	Design & Population	Intervention	NEC Findings	Notes/Interpretation
TIPP Trial [18,19]	Large RCT; 1202 ELBW infants	Indomethacin prophylaxis vs. placebo	NEC incidence: 11% (Indomethacin) vs. 10% (Placebo), *p* = 0.53	Prophylactic indomethacin did not increase the risk of NEC in this large cohort.
BeNeDuctus Trial [22]	Noninferiority RCT; <28 weeks GA; PDA >1.5 mm	Early ibuprofen vs. expectant management	NEC (Bell Stage ≥ IIa): 15.3% (Ibuprofen) vs. 17.6% (Expectant)	Expectant management was noninferior regarding NEC incidence (risk difference −2.3%).
NICHD-PDA Trial [26]	Multicenter RCT; 22–28 weeks GA; enrolled at 6–14 days	Active treatment vs. expectant management	Proven NEC: 11.3% (Active) vs. 10.0% (Expectant)	No significant difference in NEC rates, though the trial was stopped early for futility and safety.
Sung et al. Trial [23]	Single-center RCT; GA 23–30 weeks	Oral ibuprofen vs. nonintervention	NEC (Bell Stage ≥ IIb): 10% (Ibuprofen) vs. 4% (Nonintervention)	No significant difference (adjusted OR 2.52, 95% CI 0.60–10.52).
PDA- TOLERATE Trial [24]	Exploratory RCT; <28 weeks GA; enrolled at 6–14 days	Early routine treatment (ERT) vs. conservative (CT)	NEC incidence: 16% (ERT) vs. 19% (CT)	No significant benefit for NEC was found with early routine pharmacological closure.
TRIOCAPI Trial [21]	Double-blind RCT; <28 weeks GA; screened early	Early targeted ibuprofen vs. placebo	NEC (Bell Stage ≥ IIb): 4.4% (Ibuprofen) vs. 5.3% (Placebo)	Observed no significant difference between targeted ibuprofen and a strategy relying on rescue therapy.

Abbreviations: RCT (randomized controlled trial); ELBW (extremely low birth weight); NEC (necrotizing enterocolitis); GA (gestational age); PDA (patent ductus arteriosus); TIPP (Trial of Indomethacin Prophylaxis in Preterms); BeNeDuctus (Expectant Management or Early Ibuprofen for Patent Ductus Arteriosus Trial); NICHD-PDA (National Institute of Child Health and Human Development Patent Ductus Arteriosus Trial); OR (odds ratio); CI (confidence interval); ERT (early routine treatment); CT (conservative treatment).

**Table 4:** Bronchopulmonary Dysplasia Outcomes in Randomized Trials and Cohort Studies of PDA Management in Preterm Infants.

Trial	Design & Population	Intervention	BPD Findings	Notes/Interpretation
TIPP Trial [18,19]	Ancillary analysis of TIPP cohort; 999 ELBW survivors	Indomethacin prophylaxis vs. placebo	BPD incidence: 45% (Indomethacin) vs. 43% (Placebo)	Indomethacin increased BPD risk specifically in infants without a PDA (43% vs. 30%, *p* = 0.015), possibly due to adverse drug effects on oxygenation and fluid retention.
BeNeDuctus Trial [22]	Noninferiority RCT; <28 weeks GA	Early ibuprofen vs. expectant management	Moderate-to-severe BPD: 50.9% (Ibuprofen) vs. 33.3% (Expectant), ARD −17.6%	Suggests harm associated with early ibuprofen exposure, potentially driven by drug effects on angiogenesis.
NICHD-PDA Trial [26]	Multicenter RCT; 22–28 weeks GA; enrolled at 6–14 days	Active treatment vs. expectant management	BPD incidence: 77.4% (Active) vs. 80.1% (Expectant)	No significant difference in BPD rates (adjusted risk difference −2.20%).
Baby OSCAR Trial [33]	Multicenter RCT; 23–28 weeks GA; large PDA	Selective early ibuprofen vs. placebo	Death or BPD: 69.2% (Ibuprofen) vs. 63.5% (Placebo)	Noted a non-significant trend toward more BPD in the ibuprofen treatment arm.
Sung et al. Trial [23]	Single-center RCT; GA 23–30 weeks	Oral ibuprofen vs. nonintervention	BPD incidence: 45% (Ibuprofen) vs. 40% (Nonintervention)	Nonintervention was noninferior to ibuprofen for BPD or death.
PDA- TOLERATE Trial [24]	Exploratory RCT; <28 weeks GA; enrolled at 6–14 days	Early routine treatment (ERT) vs. conservative (CT)	BPD incidence: 49% (ERT) vs. 53% (CT)	Early treatment showed no significant benefit in reducing BPD risk.

Abbreviations: RCT (randomized controlled trial); ELBW (extremely low birth weight); BPD (bronchopulmonary dysplasia); PDA (patent ductus arteriosus); GA (gestational age); TIPP (Trial of Indomethacin Prophylaxis in Preterms); BeNeDuctus (Expectant Management or Early Ibuprofen for Patent Ductus Arteriosus Trial); NICHD-PDA (National Institute of Child Health and Human Development Patent Ductus Arteriosus Trial); ARD (absolute risk difference); ERT (early routine treatment); CT (conservative treatment).

**Table 5:** Kidney Injury Outcomes in Randomized Trials and Cohort Studies of PDA Management in Preterm Infants.

Trial	Design & Population	Intervention	Kidney Injury Findings	Notes/Interpretation
TIPP Trial [18,19]	Large multicenter RCT; 1202 ELBW infants	Indomethacin prophylaxis vs. placebo	Oliguria as reason for withholding dose: 7% (Indomethacin) vs. 4% (Placebo)	Indomethacin prophylaxis significantly diminished urine output in the first four days of life.
BeNeDuctus Trial [22]	Noninferiority RCT; <28 weeks GA	Early ibuprofen vs. expectant management	Renal failure incidence: 9.5% (Ibuprofen) vs. 9.6% (Expectant)	Rates of renal failure were nearly identical between the intervention and non-intervention groups.
TRIOCAPI Trial [21]	Double-blind RCT; <28 weeks GA	Early targeted ibuprofen vs. placebo	Renal failure: 12.3% (Ibuprofen) vs. 14.0% (Placebo)	No significant difference in renal failure (defined as creatinine >150 μmol/L or oliguria) was observed.
Sung et al. Trial [23]	Single-center RCT; GA 23–30 weeks	Oral ibuprofen vs. nonintervention	Oliguric renal failure: 11% (Ibuprofen) vs. 8% (Nonintervention)	Defined as urine output <0.5 mL/kg/d plus creatinine ≥2.0 mg/dL; findings were not significantly different.
DETECT Trial [20]	Early targeted RCT; <29 weeks GA	Indomethacin vs. placebo	Creatinine >150 μmol/L: 2% (Indomethacin) vs. 2% (Placebo)	Targeted early treatment was not associated with an increase in significant renal impairment.
PDA- TOLERATE Trial [24]	Exploratory RCT; <28 weeks GA	Early routine treatment (ERT) vs. conservative (CT)	Received furosemide ≥14 days: 35% (ERT) vs. 46% (CT), *p* = 0.10	Noted a non-significant trend toward less diuretic use in the treatment group.

Abbreviations: RCT (randomized controlled trial); ELBW (extremely low birth weight); GA (gestational age); Oliguria (low urine output); μmol/L (micromoles per liter); mL/kg/d (milliliters per kilogram per day); ERT (early routine treatment); CT (conservative treatment).

**Table 6:** Pulmonary Hypertension Outcomes in Randomized Trials and Cohort Studies of PDA Management in Preterm Infants.

Trial	Design & Population	Intervention	Pulmonary Hypertension Findings	Notes/Interpretation
TIPP Trial [18,19]	Large multicenter RCT; 1202 ELBW infants (500–999 g)	Indomethacin prophylaxis vs. placebo	No reports of severe pulmonary hypertension were found in infants randomized to indomethacin prophylaxis.	Conducted a specific review of the database for PH after other reports suggested a link between cyclooxygenase inhibitors and increased pulmonary vascular resistance.
TRIOCAPI Trial [21]	Double-blind RCT; <28 weeks GA; large PDA at 6–12 h	Early targeted ibuprofen vs. placebo	PH incidence: 3.5% (Ibuprofen) vs. 4.4% (Placebo), *p* = 0.74.	Observed no significant difference in PH rates between targeted early ibuprofen and a strategy relying on rescue therapy.
NICHD-PDA Trial [26]	Multicenter RCT; 22–28 weeks GA; enrolled at 6–14 days	Active treatment vs. expectant management	Active Group: 1 neonatal death from PH; Expectant Group: 0 fatal cases (but 2 deaths post-36 weeks with PH).	The trial was stopped early for futility; investigators noted potential risks of vascular toxicity with active treatment.
DETECT Trial [20]	Early targeted RCT; <29 weeks GA; screened before 12 h	Indomethacin vs. placebo for large PDA	Excluded 6 infants at screening due to evidence of raised pulmonary pressures (right-to-left shunting).	Protocol required PH to resolve by 12 h before randomization could occur, focusing the study on left-to-right shunts.
The PDA RCT [22]	Single-center pilot RCT; <29 weeks GA; high severity score	Early targeted ibuprofen vs. placebo	1 case of pulmonary hypertension occurred in the ibuprofen group, requiring early discontinuation of treatment.	PH was defined as bidirectional shunting across the PDA and was an explicit exclusion criterion at screening.
BeNeDuctus Trial [22]	Noninferiority RCT; <28 weeks GA; PDA >1.5 mm	Early ibuprofen vs. expectant management	Excluded infants with persistent pulmonary hypertension (transductal right-to-left shunt during ≥33% of the cycle).	Similar to other trials, patients with existing PH were excluded to ensure the safety of administering ibuprofen, which may increase pulmonary vascular resistance.

Abbreviations: GA (gestational age); PDA (patent ductus arteriosus); PH (pulmonary hypertension); RCT (randomized controlled trial); ELBW (extremely low birth weight).

**Table 7:** Summarized results of recent RCTs.

Trial	Trial Design & Population	Primary Outcome	Primary Outcome Results	Secondary Outcomes
Baby OSCAR Trial [33]	Multicenter, RCT, placebo-controlled; infants 23 + 0–28 + 6 wks GA with large PDA (>1.5 mm)	Death or moderate/severe BPD at 36 wks PMA	No significant difference (Ibuprofen 69.2% vs. Placebo 63.5%; aRR 1.09 [95% CI 0.98–1.2])	No difference in 2-year neurodevelopmental or respiratory outcomes; trend toward more BPD in treatment group
BeNeDuctus Trial [22]	Multicenter RCT (non-inferiority); infants <28 wks GA with PDA >1.5 mm at 24–72 h	NEC (≥Stage 2a), moderate/severe BPD, or death at 36 wks PMA	Expectant management noninferior (46.3% vs. 63.5%, aRD −17.2%; *p* < 0.001)	Higher BPD rate in treatment group (51% vs. 33%); sex-based differences observed; acetaminophen used in both arms
TRIOCAPI Trial [21]	Double-blind RCT in 11 NICUs; <28 wks GA, large PDA by echo at 6–12 h	Survival without CP at 24 months CA	No difference (Ibuprofen 71.3% vs. Placebo 71.6%; aRR 0.98 [95% CI 0.83–1.16])	High rescue rate in placebo (62%); no differences found in IVH, ventilation duration, or GI outcomes
The PDA RCT [49]	Pilot RCT using PDA severity score ≥5; <29 wks GA	CLD and/or death before discharge	No significant difference (Ibuprofen 53% vs. Placebo 60%; OR 0.8 [95% CI 0.3–2.1])	Feasibility study; noted a non-significant trend toward higher mortality in the ibuprofen group (28% vs. 13%)
Sung et al. Trial [23]	Single-center, RCT (non-inferiority); GA 23–30 wks, PDA >1.5 mm + resp. support; randomization on days 6–14	BPD or death	Nonintervention noninferior (44% vs. 50%; RD 6% [95% CI −0.11 to 0.22]; *p* = 0.51)	Improved PDA closure at 1 week; no differences in closure at discharge, NEC, ROP, or IVH
TIPP Trial [19]	Multicenter, double-blind RCT; ELBW infants (500–999 g); indomethacin x3 days	Death, CP, cognitive delay, deafness, blindness at 18 mo CA	No difference (47% vs. 46%)	Significant reduction in severe IVH and PDA rates; no difference in long-term CLD, NEC, or ROP
PDA- TOLERATE Trial [24]	Multicenter exploratory RCT; <28 wks GA, PDA at 6–14 d; ERT vs. conservative with rescue	PDA at discharge or need for ligation	No significant difference (ERT 32% vs. CT 39%)	High rescue rate (48%); ERT associated with delayed feeding and increased sepsis/death in infants ≥26 weeks
NICHD-PDA Trial [26]	Multicenter RCT; 22–28 wks GA with protocol-defined PDA, enrolled at 6–14 days of age (*n* = 482); active vs. expectant treatment	Death or BPD at 36 wks PMA	No significant difference between groups; high incidence in both arms (expectant 80.9% vs. active 79.6%) → trial stopped early for futility	Significantly higher survival and fewer fatal infections with expectant management; no reduction in BPD

Abbreviations: BPD (bronchopulmonary dysplasia); PDA (patent ductus arteriosus); GA (gestational age); RCT (randomized controlled trial); PMA (postmenstrual age); NEC (necrotizing enterocolitis); aRR (adjusted relative risk); aRD (adjusted risk difference); OR (odds ratio); CI (confidence interval); CP (cerebral palsy); CA (corrected age); ELBW (extremely low birth weight); CLD (chronic lung disease); IVH (intraventricular hemorrhage); GI (gastrointestinal); ROP (retinopathy of prematurity); ERT (early routine treatment); CT (conservative treatment).

**Table 8:** Summary of Recent PDA Trials and Analyses in Infants <26 Weeks of Gestation.

Study/ Analysis	Population Focus (<26 wks GA)	Key Findings on Outcomes	Conclusion/ Implication
Baby OSCAR Trial [33]	~50% of 653 infants <26 wks GA (≈300 infants)	Reported outcomes separately for <26 wks; included in subgroup analyses	No benefit; suggests increased risk in <26 wks subgroup
BeNeDuctus Trial [22]	56 infants (<26 wks) in Expectant group, 64 in Early Ibuprofen group (total = 120)	Subgroup analysis showed −21.2% absolute risk difference (95% CI −10.5)—higher risk with early ibuprofen	Early treatment associated with increased harm
Meta-Analysis (7 RCTs since 2010) [51]	707 infants <26 wks GA	Early NSAIDs ↑ mortality (OR 1.33, *p* = 0.05), ↑ sepsis, PVL, trends toward ↑ BPD, pulmonary hemorrhage, IVH	No benefit; signals potential harm
Review by Gupta & Donn [33]	Data from 4 trials (El-Khuffash RCT, TRIOCAPI, BeNeDuctus, Baby OSCAR)	Active treatment ↑ mortality (OR 1.25, 95% CI 1.01–1.56), worse composite death/BPD outcome (*p* = 0.009), ↑ death in <29 wks (16.2% vs. 12.0%)	Higher risk of death with pharmacological treatment
2025 Meta-Analysis (10 RCTs, 2035 infants <33 wks GA) [2]	Subgroup analysis for infants <29 wks GA	Moderate evidence of harm for BPD or death with medications (Bayes factor ≈ 0.14)	Expectant management associated with better survival and BPD outcomes
Bayesian Meta-Analysis [52]	Subgroup of infants ≤26 wks GA	Death, CP, cognitive delay, deafness, blindness at 18 mo CA	Strong/moderate Bayesian evidence against early pharmacologic PDA closure
Long-Term Follow-Up: Baby OSCAR [45], BeNeDuctus [53], TRIOCAPI	Infants followed to 24 months	No difference in survival without NDI at 24 months	No long-term benefit of early PDA closure
NICHD-PDA Trial [26]	273/481 infants (56.8%) born at 22–26 weeks GA	No difference between expectant vs. active treatment in death or BPD at 36 wks; no benefit even among infants with the largest PDAs	Active PDA treatment does not improve death or BPD at 26 wks PMA in extremely preterm infants, including those with large PDAs

Abbreviations: GA (gestational age); PDA (patent ductus arteriosus); RCT (randomized controlled trial); NSAIDs (nonsteroidal anti-inflammatory drugs); OR (odds ratio); CI (confidence interval); BPD (bronchopulmonary dysplasia); PVL (periventricular leukomalacia); IVH (intraventricular hemorrhage); CP (cerebral palsy); CA (corrected age); NDI (neurodevelopmental impairment).

## Data Availability

No new data were created.

## Abbreviations

The following abbreviations are used in this manuscript:

ACA	Anterior cerebral artery
Ao	Aorta
ASD	Atrial septal defect
LA	Left atrium
LV	Left ventricle
L→R	Left-to-right shunting direction
LVOT	Left ventricular outflow tract
MCA	Middle cerebral artery
MV	Mitral valve
PFO	Patent foramen ovale
RA	Right atrium
RV	Right ventricle
PVR	Pulmonary vascular resistance
SVR	Systemic vascular resistance
V/Q	Ventilation–perfusion mismatch
BPD	Bronchopulmonary dysplasia
CLD	Chronic lung disease
CP	Cerebral palsy
IVH	Intraventricular hemorrhage
PIVH	Papillary/intraventricular hemorrhage
NEC	Necrotizing enterocolitis
NDI	Neurodevelopmental impairment
PH	Pulmonary hemorrhage/Pulmonary hypertension
PVL	Periventricular leukomalacia
ROP	Retinopathy of prematurity
IUGR	Intrauterine growth restriction
RDS	Respiratory distress syndrome
CW	Continuous-wave Doppler
PW	Pulsed-wave Doppler
E/A	Early-to-late diastolic filling velocity ratio
ECG	Electrocardiogram
EF	Ejection fraction
SV	Stroke volume
VTI	Velocity time integral
Qp:Qs	Pulmonary-to-systemic flow ratio
FiO_2_	Fraction of inspired oxygen
cm/mm	Centimeter/Millimeter
bpm	Beats per minute
Hz/MHz	Hertz/Megahertz
μmol/L	Micromoles per liter
mL/kg/d	Milliliters per kilogram per day
GA	Gestational age
PMA	Postmenstrual age
CA	Corrected age
ELBW	Extremely low birth weight (500–999 g)
NSAID	Nonsteroidal anti-inflammatory drug
CI	Confidence interval
OR	Odds ratio
aRR	Adjusted relative risk
aRD/ARD	Adjusted/Absolute risk difference
CPAP	Continuous positive airway pressure
BeNeDuctus	Expectant Management or Early Ibuprofen for PDA (Belgium-Netherlands Trial)
TIPP	Trial of Indomethacin Prophylaxis in Preterms
TRIOCAPI	Trial on Ibuprofen in Closure of Arterial Patent Ductus
NICHDPDA	NICHD Patent Ductus Arteriosus Trial
DETECT	Australian trial on early echo screening + indomethacin
Baby OSCAR	Selective early treatment of large PDA
PDA-TOLERATE	Exploratory trial of treatment for moderate-to-large PDA at one week
BTM	Betamethasone
DART	Dexamethasone: A Randomized Trial (10-day taper)
PGE_2_	Prostaglandin E_2_
TPN	Total parenteral nutrition
PICC	Peripherally inserted central catheter
